# Olfactory impairment in the rotenone model of Parkinson’s disease is associated with bulbar dopaminergic D2 activity after REM sleep deprivation

**DOI:** 10.3389/fncel.2014.00383

**Published:** 2014-12-01

**Authors:** Lais S. Rodrigues, Adriano D. S. Targa, Ana Carolina D. Noseda, Mariana F. Aurich, Cláudio Da Cunha, Marcelo M. S. Lima

**Affiliations:** ^1^Laboratório de Neurofisiologia, Departamento de Fisiologia, Universidade Federal do Paraná, Setor de Ciências BiológicasCuritiba, Brazil; ^2^Laboratório de Fisiologia e Farmacologia do Sistema Nervoso Central, Departamento de Farmacologia, Universidade Federal do ParanáCuritiba, Brazil

**Keywords:** olfactory bulb, olfactory discrimination, dopamine, bulbar dopaminergic D2 receptors, REM sleep deprivation, intranigral, Parkinson’s disease

## Abstract

Olfactory and rapid eye movement (REM) sleep deficits are commonly found in untreated subjects with a recent diagnosis of Parkinson’s disease (PD). Additionally, different studies report declines in olfactory performance during a short period of sleep deprivation. Mechanisms underlying these clinical manifestations are poorly understood, and impairment of dopamine (DA) neurotransmission in the olfactory bulb and the nigrostriatal pathway may have important roles in olfaction and REM sleep disturbances. Therefore, we hypothesized that modulation of the dopaminergic D2 receptors in the olfactory bulb could provide a more comprehensive understanding of the olfactory deficits in PD and REM sleep deprivation (REMSD). We decided to investigate the olfactory, neurochemical, and histological alterations generated through the administration of piribedil (a selective D2 agonist) or raclopride (a selective D2 antagonist) within the glomerular layer of the olfactory bulb, in rats subjected to intranigral rotenone and REMSD. Our findings provide evidence of the occurrence of a negative correlation (*r* = −0.52, *P* = 0.04) between the number of periglomerular TH-ir neurons and the bulbar levels of DA in the rotenone, but not sham, groups. A significant positive correlation (*r* = 0.34, *P* = 0.03) was observed between nigrostriatal DA levels and olfactory discrimination index (DI) for the sham groups, indicating that increased DA levels in the substantia nigra pars compacta (SNpc) are associated with enhanced olfactory discrimination performance. Also, increased levels in bulbar and striatal DA were induced by piribedil in the rotenone control and rotenone REMSD groups, consistent with reductions in the DI. The present evidence reinforce the idea that DA produced by periglomerular neurons, particularly the bulbar dopaminergic D2 receptors, is an essential participant in olfactory discrimination processes, as the SNpc, and the striatum.

## Introduction

Olfactory deficits in odor detection (the threshold or the perception of odors at low concentrations), identification (the ability to name an odor), and discrimination (the nonverbal distinction of different smells) are commonly found in untreated subjects with a recent diagnosis of Parkinson’s disease (PD; Tissingh et al., 2001). These early dysfunctions are supported by neuropathologic studies, with Lewy pathology present in the olfactory bulb, olfactory tract and anterior olfactory nucleus in preclinical Braak stages prior to significant nigral degeneration (Del Tredici et al., 2002). Olfaction is impaired in approximately 90% of early-stage PD cases and can precede the onset of motor symptoms by years (Doty, 2012b).

It is reported a massive presence of dopaminergic inter-neurons in the glomerular portion of the olfactory bulb and the outer plexiform layer (Liberia et al., 2012). In PD the number of tyrosine hydroxylase immunoreactive periglomerular neurons (TH-ir) is increased with respect to age-matched controls, possibly reflecting a higher dopaminergic activity (Huisman et al., 2004; Mundiñano et al., 2011). Such effect in the olfactory bulb could lead to a suppression of olfactory information, particularly due to the inhibitory effect of dopamine (DA; Huisman et al., 2004), mediated by D2 receptors activity, on the transmission between olfactory receptor cells and mitral cells within the olfactory glomeruli (Sengoku et al., 2008), thus, promoting the hyposmia (Doty and Risser, 1989; Koster et al., 1999).

Rotenone exposure in rodents provides an interesting model for studying mechanisms of toxin-induced dopaminergic neuronal injury (Betarbet et al., 2000; Greenamyre et al., 2003; Segura Aguilar and Kostrzewa, 2004; Drolet et al., 2009; Dos Santos et al., 2013). In this model, a massive inhibition of mitochondrial complex I produces selective degeneration of the dopaminergic nigrostriatal system and reproduces key pathological features of clinical PD (Sherer et al., 2003; Alam and Schmidt, 2004; Dos Santos et al., 2013). The pathophysiology of olfactory loss in PD is unclear. Neuropathologic changes in (1) the olfactolry bulb and the anterior olfactory nucleus in the early disease; and (2) the olfactory cortex and limbic structures in advances stages may both be involved (Benarroch, 2010). Hyposmia has been related to nigrostriatal denervation in the early stages of PD (Siderowf et al., 2005), whereas cholinergic deficits in the limbic cortex may occur in later stages (Bohnen et al., 2010). Thus, the use of intranigral rotenone, as a model of PD, becomes attractive because it is strongly associated to the presymptomatic state of PD (Moreira et al., 2012; Dos Santos et al., 2013).

Another intriguing prodromal dysfunction found in PD concerns the rapid eye movement (REM) sleep disorders (Lima et al., 2012; Lima, 2013). Several findings demonstrated that normal REM sleep can be suppressed in both normal and DA transporter knockout mice without affecting motor functions by diminishing dopaminergic tone (Dzirasa et al., 2006; Lima, 2013). A different study reported that the selective D2 blockade may produce the reduction or even suppression of REM sleep after a period of REM sleep deprivation (REMSD; Lima et al., 2008; Lima, 2013). Also, the lesion of the substantia nigra pars compacta (SNpc) dopaminergic neurons provoked a major impairment in REM sleep generation in rats (Lima et al., 2007) and rhesus monkeys (Barraud et al., 2009). Interestingly, different studies have shown that REMSD, both in humans and rodents, is capable to produce a significant impairment in an olfactory discrimination task (ODT), similar to PD (Greiner et al., 2001; Fantini et al., 2006; Killgore and Mcbride, 2006; Killgore et al., 2008a, 2010.

These observations, together with recent evidence based on the use of the intranigral rotenone model of PD (Moreira et al., 2012; Dos Santos et al., 2013), have led to the hypothesis that a modulation of the D2 receptors in the olfactory bulb could provide a more comprehensive understanding of the olfactory deficits mechanisms found in PD and after REMSD. In this study, we decided to investigate the olfactory, neurochemical and histological alterations generated by the administration of piribedil or raclopride (selective D2 agonist and antagonist, respectively), within the glomerular layer of the olfactory bulb, in rats submitted to intranigral rotenone infusion. Concurrently, we evaluated the effects of REMSD in the present experimental paradigm.

## Material and methods

### Ethics statement

The studies were conducted in accordance with the guidelines of Brazilian Guide for Care and Use of Laboratory Animals (COBEA). In addition, the protocol complies with the recommendations of Federal University of Paraná and was approved by the Institutional Ethics Committee (approval ID #658).

### Animals

Male Wistar rats from our breeding colony weighing 280–320 g were used. The animals were randomly housed in groups of five in polypropylene cages with wood shavings as bedding and maintained in a temperature-controlled room (22 ± 2°C) on a 12-h light-dark cycle (lights on at 7:00 AM). The animals had free access to water and food throughout the experiment.

### Experimental design

Before the stereotaxic surgeries the animals were distributed randomly in two groups: Sham (*n* = 102) and Rotenone (*n* = 102). After the surgery procedure, the animals were redistributed in twelve groups (*n* = 17/group): sham control vehicle, sham control piribedil, sham control raclopride, sham REMSD vehicle, sham REMSD piribedil, sham REMSD raclopride, rotenone control vehicle, rotenone control piribedil, rotenone control raclopride, rotenone REMSD vehicle, rotenone REMSD piribedil, rotenone REMSD raclopride. The concentrations of these drugs were determined according to a concentration-effect curve previously defined in an experiment based on locomotor and olfactory parameters (see supplementary material, Figure 1). In addition, regarding the olfactory discrimination evaluation, we included another control group (*n* = 12) treated with Zicam (Matrixx Initiatives, Inc., Phoenix, AZ) intending to produce loss of olfaction (Lim et al., 2009).

Stereotaxic surgeries were performed on day zero and on day 4 all animals were kept individually in their home cages for 48 h to collect sawdust for the ODT. On day 6, the REMSD procedure started. Twenty four hours after, on day 7, the rats received the intrabulbar administration of piribedil or raclopride, subsequently (2 h after) they were tested (due to pharmacokinetics). Without delay, after the ODT, 10–12 animals/group were decapitated, their brains removed and dissections of the striatum, substantia nigra and olfactory bulb were subsequently used for neurochemistry and molecular analyses. Others 3–5 animals/group had their brains perfused and fixed for subsequent immunohistochemical analysis.

### Stereotaxic surgery

Rats were sedated with intraperitoneal xylazine (10 mg/kg; Syntec do Brasil Ltda, Brazil) and anesthetized with intraperitoneal ketamine (90 mg/kg; Syntec do Brasil Ltda, Brazil). The following coordinates were used to the bilateral injury, bregma as a reference: SNpc (AP) = −5,0 mm, (ML) = ± 2,1 mm e (DV) = −8,0 mm (Paxinos and Watson, 2005). Needles were guided to the region of interest for a bilateral infusion of 1 μL of rotenone (12 μg/μL) or of dimethylsulfoxide—DMSO (Sigma-Aldrich®, United States) using an electronic infusion pump (Insight Instruments, Ribeirão Preto, Brazil) at a rate of 0.33 μL/min for 3 min (Saravanan et al., 2005; Moreira et al., 2012; Dos Santos et al., 2013). Complementarily, a guide cannula was implanted in the olfactory bulb of each rat allowing a subsequent 1 μL infusion of piribedil (3 μg/μL) (Tocris Bioscience®, United Kingdom), raclopride (10 μg/μL) (Sigma-Aldrich®, United States) or vehicle (DMSO) at a rate of 0.33 μL/min for 3 min, in their respective groups. Coordinates with reference to bregma for implantation of guide cannula were: (AP) = +7.08 mm (ML) = 0.0 mm and (DV) = −3.6 mm (Paxinos and Watson, 2005).

### Intranasal administration of zicam (zinc gluconate + zinc acetate solution)

The administration of Zicam® Oral Mist (Matrixx Initiatives, Scottsdale, AZ, USA) was performed as previously reported by (Chioca et al., 2013): “the animals were sedated with an intraperitoneal administration of 90 mg/kg ketamine and 3 mg/kg xylazine, and approximately 30 μL of Zicam solution was slowly delivered into the nasal cavity using a Hamilton syringe connected to a blunted 30-gauge needle through a polyethylene tube. The needle was inserted 15 mm past the right external nostril to help irrigate the olfactory epithelium. The procedure was repeated in the left nostril. During respiration, part of the solution was expelled through the nostril and dried with absorbent paper to allow the animal to continue breathing”.

### REMSD procedure

REMSD was attained by means of the single platform method. Rats were individually placed on a circular platform (6.5 cm in diameter) in a cage (23 × 23 × 30 cm) filled with water up to 1 cm below the platform level. At the onset of each REM sleep episode, the animal experiences a loss of muscle tonus and falls into the water, thus being awakened. When platforms of this size are used, REM sleep is completely eliminated (Machado et al., 2004). Throughout the study, the experimental room was maintained at controlled conditions (22 ± 2°C, 12 h light/dark cycle, lights on 7:00 a.m.). The control group was kept in the same room as the REMSD rats during the study. Food and water were provided *ad libitum* by placing chow pellets in a dispenser positioned inside the cage and water bottles on a grid located on top of the tank.

### Olfactory discrimination task (ODT)

This test was previously described by Lamberty Soffie and subsequently modified by Prediger et al. (Soffié and Lamberty, 1988; Prediger et al., 2005a,b). The version used has been modified from (Prediger et al., 2005a). The apparatus consists of a box (60 × 40 × 50 cm) equally divided into two compartments, connected by a door that gives free access to the animal. Before the test, the animals were allowed an adaptation period in the apparatus of 5 min, in both compartments with fresh sawdust. After that, clean sawdust is added on one side of the box (non-familiar odor). Sawdust obtained from cages of animals isolated for 48 h before testing was added to the other side of the box (familiar odor). The ODT consists of placing the rat in the middle of olfactory discrimination box and recording, up to 5 min, the time of investigation of each compartment. The animal that shows olfactory impairment tends to explore both compartments equally, indicating absence of discrimination. As a parameter of discrimination, a “discrimination index (DI)” was considered by dividing the difference in exploration time between the two compartments (compartment non-familiar—compartment familiar) by the total amount of exploration for both compartments (compartment non-familiar + compartment familiar). DI was then multiplied by 100 to express it as a percentage (Dos Santos et al., 2013).

### Neurochemical analysis of striatal, olfactory bulb and substantia nigra neurotransmitters, metabolites and turnovers

The brains were quickly removed from the skulls after decapitation (about 30 s long) then were immediately placed on petri dishes (covered with a filter paper) immersed on dry ice. The striatum, olfactory bulb and the substantia nigra of the rats were immediately dissected and placed on pre-weighed eppendorf tubes that were weighed again with the structures. As previously reported by (Moreira et al., 2012): “the samples were stored at −80°C until analysis. The endogenous concentrations of DA, 3,4-dihydroxyphenylacetic acid (DOPAC), homovanillic acid (HVA) were assayed by reverse-phase high performance liquid chromatography (HPLC) with electrochemical detection (Barbiero et al., 2011). The system consisted of a Synergi Fusion-RP C-18 reverse-phase column (150 × 4.6 mm i.d., 4 μm particle size) fitted with a 4 × 3.0 mm pre-column (Security Guard Cartridges Fusion-RP); an electrochemical detector (ESA Coulochem III Electrochemical Detector) equipped with a guard cell (ESA 5020) with the electrode set at 350 mV and a dual electrode analytical cell (ESA 5011A); a LC-20AT pump (Shimadzu) equipped with a manual Rheodyne 7725 injector with a 20 μL loop. The column was maintained inside in a temperature-controlled oven (25°C). The cell contained two chambers in series: each chamber including a porous graphite coulometric electrode, a double counter electrode and a double reference electrode. Oxidizing potentials were set at 100 mV for the first electrode and at 450 mV for the second electrode. The tissue samples were homogenized with an ultrasonic cell disrupter (Sonics) in 0.1 M perchloric acid containing sodium metabisulfite 0.02% and internal standard. After centrifugation at 10,000 × *g* for 30 min at 4°C, 20 μL of the supernatant was injected into the chromatograph”.

“The mobile phase, used at a flow rate of 1 mL/min, had the following composition: 20 g citric acid monohydrated (Merck), 200 mg octane-1-sulfonic acid sodium salt (Merck), 40 mg ethylenediaminetetraacetic acid (EDTA) (Sigma), 900 mL HPLC-grade water. The pH of the buffer running solution was adjusted to 4.0 then filtered through a 0.45 μm filter. Methanol (Merck) was added to give a final composition of 10% methanol (v/v). The neurotransmitters and metabolites concentrations were calculated using standard curves that were generated by determining in triplicate the ratios between three different known amounts of the internal standard. The unit was expressed as ng/g of wet weight”.

### TH- immunohistochemistry within the olfactory bulb

Total number of TH-ir neurons was estimated in the glomerular and plexiform layers of the olfactory bulb (Figure 5C). Animals were deeply anesthetized with ketamine immediately after the ODT, and were intracardially perfused with saline first, then with 4% of the fixative solution formaldehyde in 0.1 M phosphate buffer (pH 7.4). Brains were removed from the skulls and were immersed for 48 h in that fixative solution at 4°C. Subsequently, the brains were placed in 30% sucrose solution for 3 days and were frozen at −80°C before sectioning. Twelve 40 μm sections per animal were taken from the olfactory bulb (+7.56 mm and +7.08 mm an interval of 480 μm). Tissue sections were incubated with primary mouse anti-TH antibody, diluted in phosphate-buffered saline containing 0.3% Triton X-100 (1:500; Chemicon, CA, USA) overnight at 4°C. Biotin-conjugated secondary antibody incubation (1:200 anti-mouse # Vector Laboratories, USA), was performed for 2 h at room temperature. After several washes in phosphate-buffered saline, antibody complex was localized using the ABC system (Vectastain ABC Elite kit, Vector Laboratories, USA) followed by 3,3′-diaminobenzidine reaction with nickel enhancement. The sections were then mounted onto gelatin-coated slides and coverslipped after dehydration in ascending concentrations of ethanol-xylene solutions. Cell counts were conducted making use of the software Image-Pro Express 6. Counts were done on 10–12 tissue sections (corresponding to the 480 μm interval), and an average count per section (and consequently for each animal) was obtained. For each group a mean value was calculated and converted to a percentage relative to the sham control vehicle, and compared with those of the other groups. The mean number of TH-ir neurons in each hemisphere was considered to be representative of the olfactory bulb neuronal cells in each animal. The images were obtained using a motorized Axio Imager Z2 microscope (Carl Zeiss, Jena, DE), equipped with a automated scanning VSlide (Metasystems, Altlussheim, DE).

**Figure 1:**
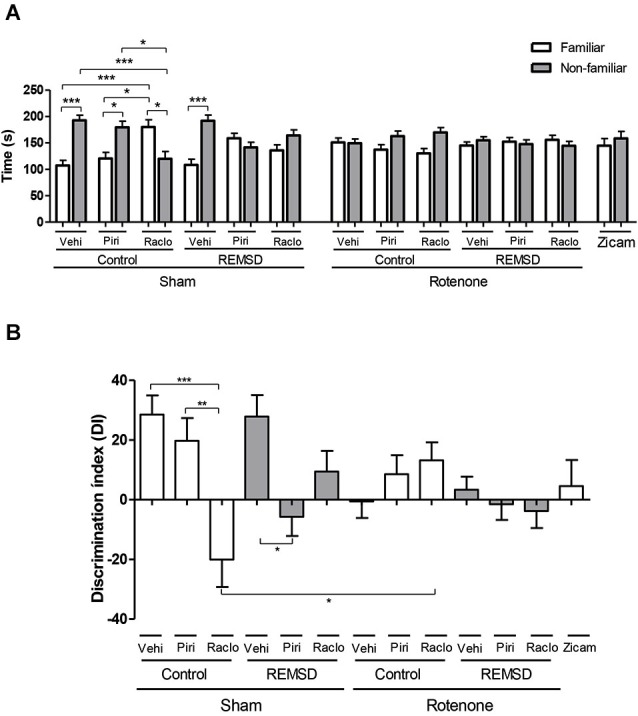
**(A)** Time (s) spent in familiar and non-familiar compartments in the olfactory discrimination task (ODT) 7 days after surgery. The bars represent the mean ± standard error of the mean. *n* = 15 per group, *P ≤ 0.05, ***P ≤ 0.001 comparing the time mean spent in familiar and non-familiar compartments. Two-way ANOVA followed by the Bonferroni test. **(B)** Olfactory discrimination index (DI) calculated by (NF-F/NF+F)*100, NF is the time spent in the compartment with non-familiar odor and F is the time spent in the compartment with familiar odor. The bars represent the mean ± standard error of the mean, *n* = 15 per group, *P ≤ 0.05, **P ≤ 0.01, ***P ≤ 0.001. One-way ANOVA followed by the Newman-Keuls test.

**Figure 2:**
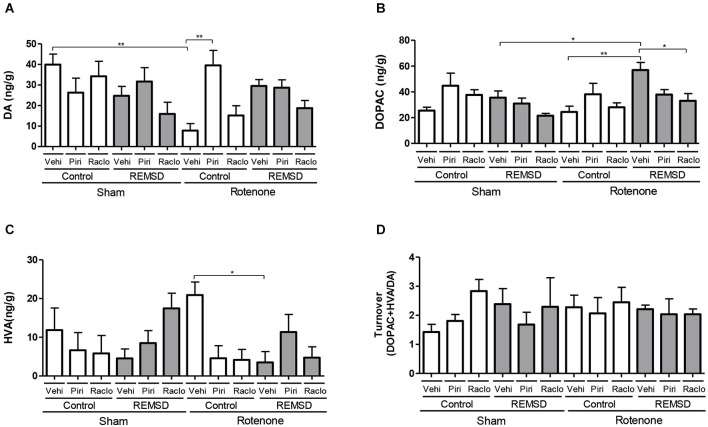
**Neurochemical examination of the olfactory bulb content of DA and metabolites. (A)** DA, **(B)** DOPAC, **(C)** HVA, **(D)** DA turnover. Values are expressed as mean ± SEM. *P ≤ 0.05, **P ≤ 0.01. One-way ANOVA followed by the Newman Keuls test.

**Figure 3:**
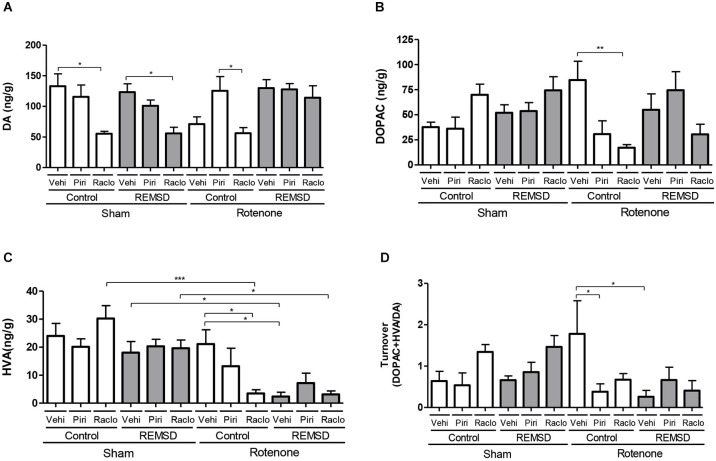
**Neurochemical examination of the substantia nigra content of DA, and metabolites. (A)** DA, **(B)** DOPAC, **(C)** HVA, **(D)** DA turnover. Values are expressed as mean ± SEM. *P ≤ 0.05, **P ≤ 0.01, ***P ≤ 0.001. One-way ANOVA followed by the Newman Keuls test.

**Figure 4:**
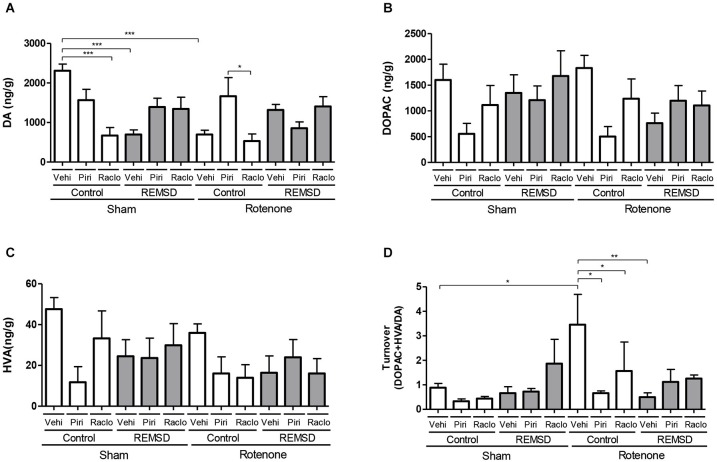
**Neurochemical examination of the striatal content of DA and metabolites. (A)** DA, **(B)** DOPAC, **(C)** HVA, **(D)** DA turnover. Values are expressed as mean ± SEM. *P ≤ 0.05, **P ≤ 0.01, ***P ≤ 0.001. One-way ANOVA followed by the Newman Keuls test.

**Figure 5:**
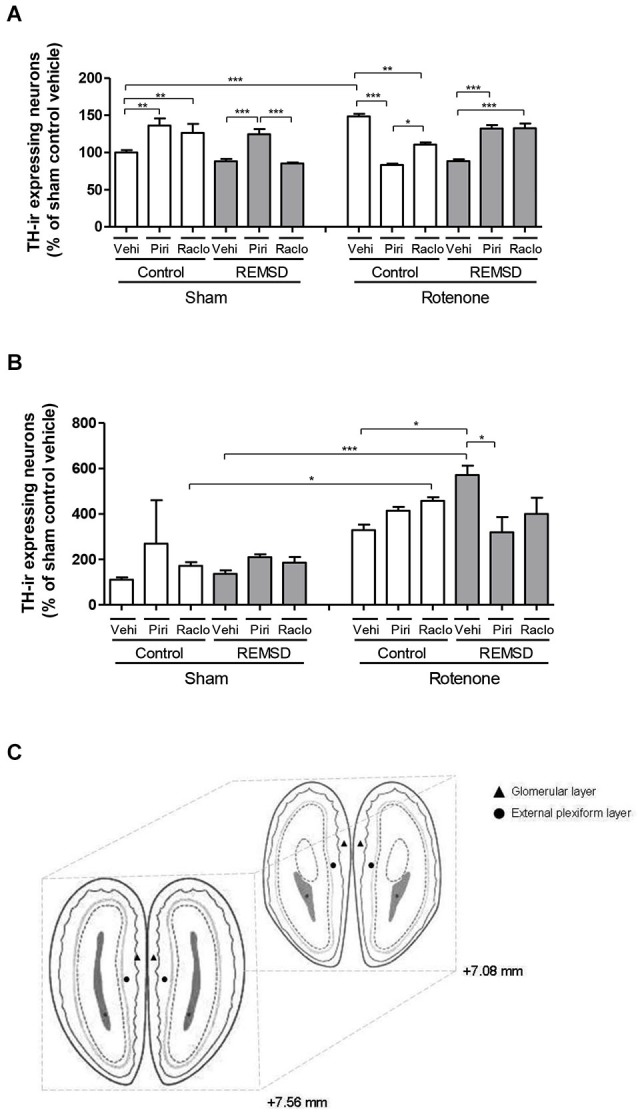
**(A)** Percentage of TH-ir expressing neurons in the glomerular layer of the olfactory bulb in relation to sham control vehicle group. The bars represent the mean ± standard error of the mean, *n* = 5 per group, *P ≤ 0.05, **P ≤ 0.01, ***P ≤ 0.001. One-way ANOVA followed by the Newman-Keuls test. **(B)** Percentage of TH-ir expressing neurons in the external plexiform layer of the olfactory bulb in relation to sham control vehicle group. The bars represent the mean ± standard error of the mean, *n* = 5 per group, *P ≤ 0.05, ***P ≤ 0.001. One-way ANOVA followed by the Newman-Keuls test. **(C)** Schematic diagram representing the coordinates (mm) and sections used in immunohistochemistry for TH in the glomerular layer (triangle) and external plexiform layer (circle) of the olfactory bulb (adapted from Paxinos and Watson, 2005).

#### Statistical analysis

Differences between groups in the ODT were analyzed by two-way analysis of variance (ANOVA) followed by the Bonferroni *post hoc* test. Olfactory DI, neurochemical analysis and TH-immunohistochemistry were analyzed by one-way ANOVA followed by the Newman-Keuls multiple comparison test. Pearson’s correlation coefficients (*r*) were calculated to establish relationships between neurochemical and behavioral parameters or molecular and neurochemical parameters. Values were expressed as mean ± standard error of mean (SEM). The level of significance was set at *P* ≤ 0.05.

## Results

### Olfactory discrimination task (ODT)

Regarding the time spent in each compartment in the ODT, we found that the sham control vehicle group showed an increment in time spent in the non-familiar odor compartment in relation to the familiar (*P* ≤ 0.001), as well as the sham control piribedil (*P* ≤ 0.05) and sham REMSD vehicle (*P* ≤ 0.001) groups (Figure 1A). In contrast, the sham control raclopride group showed more time exploring the familiar odor compartment in relation to the non-familiar (*P* ≤ 0.05) and that time spent in the familiar compartment was significantly higher when compared to the sham control piribedil group (*P* ≤ 0.05). In fact, the time spent in the non-familiar compartment for the sham control raclopride group was significantly lower in comparison to the sham control vehicle (*P* ≤ 0.001) and sham control piribedil (*P* ≤ 0.05) groups as indicated by the odor (*F*_(12,292)_ = 8.46, *P* < 0.0001), group (*F*_(12,292)_ = 0.00, *P* = 1.00) and interaction (*F*_(12,292)_ = 8.46, *P* < 0.0001) factors (Figure 1A). In addition, this analysis revealed that sham REMSD piribedil and sham REMSD raclopride did not discriminated the compartments. A similar result was obtained for all the rotenone groups, as well as for the Zicam group (positive control of olfactory loss) (Figure 1A).

Figure 1B shows the DI for each group obtained during the ODT. Accordingly, the sham control raclopride group exhibited a significant impairment in the DI compared to the sham control piribedil (*P* < 0.001) and sham control vehicle (*P* < 0.05) groups. Likewise, the sham control raclopride group demonstrated a significant reduction (*P* < 0.05) in this parameter in comparison to the rotenone control raclopride group (*F*_(12,146)_ = 4.236, *P* < 0.0001). In addition, the sham REMSD piribedil presented a reduction (*P* < 0.05) in the DI compared to the sham REMSD vehicle.

### Quantification of bulbar, nigral and striatal dopamine and metabolites

Figure 2 shows the alterations in the neurotransmission within the olfactory bulb. Accordingly, DA levels (Figure 2A) were reduced in the rotenone control vehicle group compared to the sham control vehicle (*P* ≤ 0.01) and rotenone control piribedil (*P* ≤ 0.01) groups (*F*_(11,78)_=3.678, *P* = 0.0003). Considering the DOPAC levels (Figure 2B) it was observed that the rotenone REMSD vehicle group presented an increase of this metabolite compared to the sham REMSD vehicle (*P* ≤ 0.05), rotenone control vehicle (*P* ≤ 0.01) and rotenone REMSD raclopride (*P* ≤ 0.01) groups (*F*_(11,86)_ = 3.569, *P* = 0.0004). Figure 2C shows the HVA levels in the olfactory bulb, that is increased in the rotenone control vehicle group compared to the rotenone REMSD vehicle group (*P* ≤ 0.05; *F*_(11,80)_ = 2.146, *P* = 0.0259). The calculation of the bulbar DA turnover (Figure 2D) indicated no differences between groups (*F*_(11,41)_ = 0.5327, *P* = 0.8696).

The neurochemical alterations in the substantia nigra are shown in Figure 3. DA levels (Figure 3A) were found to be reduced in the sham control raclopride group compared to the sham control vehicle group (*P* ≤ 0.05). A rather similar result is found for the sham REMSD raclopride group compared to sham REMSD vehicle group (*P* ≤ 0.05), as well as for the rotenone control raclopride group compared to rotenone control piribedil group (*P* ≤ 0.05) (*F*_(11,79)_ = 4.987, *P* < 0.0001). Regarding the DOPAC levels (Figure 3B) the rotenone control raclopride group presented a decrease in this metabolite in comparison to the rotenone control vehicle group (*P* ≤ 0.01) (*F*_(11,80)_=2.978, *P* = 0.0023). Figure 3C shows the HVA levels, that is increased in the sham control raclopride group compared to the rotenone control raclopride group (*P* ≤ 0.001). Furthermore, the sham REMSD vehicle group exhibited a significant increase in HVA compared to the rotenone REMSD vehicle group (*P* ≤ 0.05). Besides, the sham REMSD raclopride group presented an increment in HVA levels compared to the rotenone REMSD raclopride group (*P* ≤ 0.05). Likewise, the rotenone control vehicle group showed an increase in HVA compared to the rotenone REMSD vehicle (*P* ≤ 0.05) and rotenone control raclopride groups (*P* ≤ 0.05) (*F*_(11,87)_ = 6.768, *P* < 0,0001). The calculation of the nigral DA turnover (Figure 3D) indicated that the rotenone control vehicle group presented an increased turnover (*P* ≤ 0.05) compared to the rotenone control piribedil and rotenone REMSD vehicle groups (*F*_(11,42)_ = 2.907, *P* = 0,0062).

In the striatum, the neurochemical alterations are shown in Figure 4. DA levels (Figure 4A) were found to be reduced in the sham control raclopride, sham REMSD vehicle and rotenone control vehicle groups compared to the sham control vehicle group (*P* ≤ 0.001). Similar results were found for the rotenone control raclopride group compared to rotenone control piribedil group (*P* ≤ 0.05) (*F*_(11,75)_ = 5.654, *P* < 0.0001). Considering the DOPAC levels (Figure 4B) no differences among the groups (*F*_(11,75)_=1.872, *P* = 0.0567) were identified. Absence of statistical significances were also found regarding the HVA levels (*F*_(11,76)_ = 1.572, *P* = 0.1243) (Figure 4C). The calculation of the striatal DA turnover (Figure 4D) indicated that the rotenone control vehicle group presented an increase in this parameter compared to the sham control vehicle (*P* ≤ 0.05), rotenone control piribedil (*P* ≤ 0.05), rotenone control raclopride (*P* ≤ 0.05) and rotenone REMSD vehicle (*P* ≤ 0.01) groups (*F*_(11,37)_ = 2.593, *P* = 0,0149).

### TH-immunohistochemistry

As can be seen in Figure 5A, the percentage of TH-ir neurons within the glomerular layer of the olfactory bulb indicated that a significant increase in TH-ir neurons was observed in the sham control piribedil (*P* < 0.01) and sham control raclopride (*P* < 0.01) groups in comparison to the sham control vehicle group. Besides, the sham REMSD piribedil group exhibited a significant increase of this parameter compared to the sham REMSD vehicle (*P* < 0.001) and sham REMSD raclopride (*P* < 0.001) groups. The sham REMSD raclopride group also presented a decrease of TH-ir neurons compared to the sham control raclopride group (*P* < 0.001). The intranigral rotenone administration inflicted a rather opposite effect regarding the number of TH-ir neurons, i.e., the rotenone control piribedil group showed a decrease of this labeling compared to the rotenone control vehicle (*P* < 0.001) and rotenone control raclopride (*P* < 0.05). Moreover, the rotenone REMSD piribedil and rotenone REMSD raclopride groups showed increased percentage in TH-ir neurons in comparison to the rotenone REMSD vehicle group. In addition, the rotenone REMSD piribedil group demonstrated an increase in TH-ir neurons compared to the rotenone control piribedil (*P* < 0.001). Remarkably the rotenone control piribedil group exhibited a significant decrease in the percentage of TH-ir neurons in comparison to the sham control piribedil group (*P* < 0.001). In contrast, the rotenone control vehicle group showed an increment in this labeling compared to the sham control vehicle (*P* < 0.001). Moreover, a significant increase (*P* < 0.001) was identified in the rotenone REMSD raclopride group compared to the sham REMSD raclopride group (*F*_(11,28)_ = 17.80, *P* < 0.0001).

In relation to the plexiform layer of the olfactory bulb (Figure 5B) a significant increase in TH-ir neurons was observed in the rotenone control raclopride (*P* < 0.05) and rotenone REMSD vehicle (*P* < 0.001) compared to their respective sham groups. Additionally, an increment in the number of these neurons was perceived in the rotenone REMSD vehicle group compared to the rotenone control vehicle (*P* < 0.05) and rotenone REMSD piribedil (*P* < 0.05) groups (*F*_(11,30)_ = 7.504, *P* < 0.0001).

### Statistical correlations between behavioral and neurochemical parameters or molecular and neurochemical parameters

Pearson’s correlation coefficients (Table 1) revealed a moderate negative correlation (*r* = −052; *P* = 0.04) between periglomerular TH-ir neurons and bulbar DA levels for the rotenone groups. We noted a weak, however, significant positive correlation (*r* = 0.34 ; *P* = 0.03) between nigral DA levels and DI for the sham groups. Also, it has been noted a significant positive correlation (*r* = 0.30; *P* = 0.04) between striatal DA levels and DI for the rotenone groups.

**Table 1:** **Pearson’s correlations between different behavioral, neurochemical and histological parameters**.

Correlations	Groups	
	Sham	Rotenone
Periglomerular TH-ir neurons × bulbar DA	*r* = 0.24; *P* = 0.25	*r* = −052; *P* = 0.04*
Periglomerular TH-ir neurons × nigral DA	*r* = −0.21; *P* = 0.31	*r* = −032; *P* = 0.21
Periglomerular TH-ir neurons × striatal DA	*r* = −0.06; *P* = 0.76	*r* = −0.38; *P* = 0.14
Bulbar DA × DI	*r* = −0.12; *P* = 0.40	*r* = −0.10; *P* = 0.49
Nigral DA × DI	*r* = 0.34; *P* = 0.03*	*r* = −0.21; *P* = 0.15
Striatal DA × DI	*r* = 0.13; *P* = 0.37	*r* = 0.30; *P* = 0.04*

*Significant correlations are indicated.

## Discussion

In the present study, we observed that both intranigral rotenone and REMSD were able to produce olfactory dysfunction under the modulation of D2 receptors within the glomerular layer of the olfactory bulb. The extent of the olfactory deficit was moderate, considering the effects of REMSD, and strongly associated with D2 activation. However, rotenone evoked an overwhelming impairment (possibly due to a ceiling effect) because additional pharmacological manipulation did not continue to produce any difference. It is worth noting that in this study, we observed that the olfactory effects generated by rotenone were quite similar to the olfactory deficit inflicted by intranasal Zicam, which was used as a positive control for olfactory impairment. This agent has been reported to promote cytotoxicity in both mouse and human nasal tissue, including a potential for the development of long-lasting and possibly irreversible olfactory dysfunction (Lim et al., 2009; Chioca et al., 2013). The neurotoxic effects of rotenone have been typically associated with nigrostriatal dopaminergic neurotransmission (Betarbet et al., 2000; Moreira et al., 2012; Dos Santos et al., 2013); however, to the best of our knowledge, this is the first study to compare variations in monoaminergic neurotransmission within the olfactory bulb, striatum and SNpc following rotenone exposure. In fact, rotenone alone appears to reduce the level of DA in the olfactory bulb and striatum, whereas REMSD produced a similar effect in the striatum. The blockade of bulbar D2 receptors triggered a considerable suppression of DA, which was also observed after REMSD, within the SNpc and striatum. However, a notable increase in DA levels was detected in the rotenone control piribedil group in the olfactory bulb, SNpc and striatum. This increase was completely abolished in the rotenone REMSD piribedil group. Moreover, the number of olfactory TH-ir periglomerular neurons was increased in both the activated and blocked D2 receptors of the sham control groups. Conversely, only D2 activation was able to increase this labeling in the presence of REMSD. Additionally, rotenone plus REMSD led to a drastic reduction in the number of rescued TH-ir neurons, most likely by the D2 agonist piribedil. These data are in accordance with previous reports that also described the involvement of the dopaminergic system in olfaction (Mundiñano et al., 2011; Hutter and Chapman, 2013; Borghammer et al., 2014). We suppose that the increased olfactory TH-ir neurons that we observed following rotenone treatment might be the result of phenotypic and perhaps epigenetic changes in pre-existing olfactory GABAergic neurons.

Various studies have observed a decline in olfactory performance during a short period of sleep deprivation (Killgore and Mcbride, 2006; Killgore et al., 2008b, 2010). Furthermore, the relationship between dopaminergic neurotransmission and REM sleep is a recent theme in the literature, and growing evidence suggests a significant impact of PD on REM sleep disturbances (Lima, 2013). In addition, electrophysiological data indicate that the loss of half of the SNpc TH-ir neurons in rats provoked a major impairment in sleep-wake patterns, predominantly affecting REM sleep (Lima et al., 2007). In addition, REM sleep could be recovered in dopaminergic transporter knockout (DAT-KO) mice via selective activation of the D2(but not the D1) receptor, suggesting a particular role for this receptor in the regulation of REM sleep (Dzirasa et al., 2006). The involvement of DA has previously been reported following sleep deprivation protocols, indicating direct involvement in the generation of burly dopaminergic D2 supersensitivity (Tufik et al., 1978; Tufik, 1981; Nunes Júnior et al., 1994). In a similar vein, our findings provide evidence of a negative correlation (*r* = −0.52, *P* = 0.04) between the number of periglomerular TH-ir neurons and the bulbar levels of DA in the rotenone(but not the sham) group. That is, a decrease in the number of TH-ir neurons in the olfactory bulb tends to promote an increase in DA that is expressed exclusively in periglomerular cells (Hálasz et al., 1977). This effect is also associated with the olfactory deficit that is promoted by rotenone and counteracted by piribedil but not raclopride.

During a neurodegenerative disorder, periglomerular dopaminergic interneurons release DA and gamma aminobutyric acid (GABA; Mundiñano et al., 2011), which inhibit glutamatergic neurotransmission from receptor neurons to the hyperactivated mitral cells (Hsia et al., 1999; McKeith et al., 2005). Mundiñano et al. (2011) suggested that changes in centrifugal afferents to the olfactory bulb lead to changes in mitral cell activity, which are followed by compensation via an increase in the number of periglomerular dopaminergic cells to adjust for the mitral cell activity imbalance. This compensatory increase in dopaminergic inhibitory drive might underlie hyposmia in PD patients (Mundiñano et al., 2011). In accordance with these findings, a significant positive correlation (*r* = 0.34, *P* = 0.03) was observed between nigral DA and the DI in the sham groups, indicating that increased DA levels in the SNpc are associated with enhanced olfactory discrimination performance. Nevertheless, our study failed to detect possible significant negative correlations between the groups (*r* = −0.12; *P* = 0.40 for the sham groups and *r* = −0.10; *P* = 0.49 for the rotenone groups) with respect to the levels of DA in the olfactory bulb. A similar correlation (*r* = 0.30, *P* = 0.04) was identified between striatal DA and the DI in the rotenone groups.

D2 receptors are the most numerous subtype of DA receptors in the olfactory bulb (Coronas et al., 1997). It has been reported that one purposeful effect of D2 receptor activation in the olfactory bulb is a significant depression in synaptic transmission between olfactory receptor neurons and mitral cells (Hsia et al., 1999). Interestingly, we administered concentrations of piribedil and raclopride, which had been obtained from a preliminary study (supplementary material Figure 1), that increased and decreased, respectively, olfactory discrimination performance without causing locomotor bias (supplementary material Figure 2). However, this result contradicts the previous statement that a potential limitation of our data is due to the additional effect of piribedil on D3 receptors (Cagnotto et al., 1996). According to (Gutièrrez-Mecinas et al., 2005), “there is no other anatomical evidence demonstrating that D2 receptors are presynaptically located in glutamatergic synapses from the olfactory nerve onto mitral/tufted cells and periglomerular cells”. Furthermore, the occurrence of D2 receptors in the other components that form the glomerular circuitry (i.e., intraglomerular dendrites of mitral/tufted cells and periglomerular cells) has not yet been analyzed, as previously reported (Gutièrrez-Mecinas et al., 2005).

It has been verified that DA and a D2 agonist (bromocriptine) are able to modulate GABAA receptors, promoting GABAergic responses in cultured mitral and tufted cells within the rat olfactory bulb (Brünig et al., 1999). An analogous effect of DA and D2 receptors cannot be discarded in the intraglomerular dendrites of *in vivo* cells, taking into account that GABAA receptors are present in the mitral/tufted cells of the rat olfactory bulb (Giustetto et al., 1998; Panzanelli et al., 2004; Gutièrrez-Mecinas et al., 2005). If the activation of D2 receptors regulates GABAA receptors and thereby facilitates GABAergic neurotransmission from TH-ir periglomerular neurons, one would expect to observe an increase in the inhibition of mitral/tufted cells (Tillerson et al., 2006). Alternatively, the blockade of pre-synaptic D2 receptors by raclopride in olfactory receptor neurons could trigger an increase in glutamate release, consequently activating the dopaminergic juxtaglomerular neurons and increasing the levels of DA, as well as GABA, from these cells (O’Connor and Jacob, 2008; Doty, 2012a). This mechanism may induce an inhibitory effect triggered by the post-synaptic D2 and GABAA receptors that promotes olfactory impairment. It is worth noting that we observed increased levels of bulbar, nigral and striatal DA induced by piribedil in the rotenone control and rotenone REMSD groups that were consistent with the observed reduction in the DI (which was close to 3% and similar to the effect of Zicam 4.5%). It is therefore conceivable that the activation of D2 by piribedil, which is associated with the well-known dopaminergic D2 supersensitivity induced by rotenone and also by REMSD, could enhance this inhibitory effect generated by D2 receptors and thereby promote a massive impairment in olfactory function.

The present findings provide new information regarding the roles of DA and D2 receptors within the olfactory bulb, SNpc and striatum during olfactory discrimination. Rotenone promoted a remarkable level of olfactory impairment that was modulated by REMSD via D2 receptors. Indeed, D2 receptor activation can enhance or impair the ability to discriminate odors, presumably by altering the perceived intensity of a given odorant through changes in the effective sensitivity of bulbar neurons to olfactory sensory neuron input (Wei et al., 2006; Escanilla et al., 2009). DA concentrations have also been found to increase during odor learning process (Coopersmith et al., 1991), suggesting that DA modulation may play important roles in synaptic plasticity within the bulb while also influencing other areas, such as the SNpc and striatum. Therefore, the present evidence reinforces that the DA produced by periglomerular TH-ir neurons and the bulbar dopaminergic D2 receptors are essential participants in olfactory discrimination processes, as well as in the SNpc and striatum. Consequently, these changes may have a direct impact on the prodromal abnormalities found in patients with PD.

## Conflict of interest statement

The authors declare that the research was conducted in the absence of any commercial or financial relationships that could be construed as a potential conflict of interest.
