# Supplementary figures and images for: Olfactory impairment in the rotenone model of Parkinson’s disease is associated with bulbar dopaminergic D2 activity after REM sleep deprivation
Source: Front Cell Neurosci. 2014 Dec 1;8:383. doi: 10.3389/fncel.2014.00383 (PMC4249459; doi:10.3389/fncel.2014.00383)

Glomerular layer

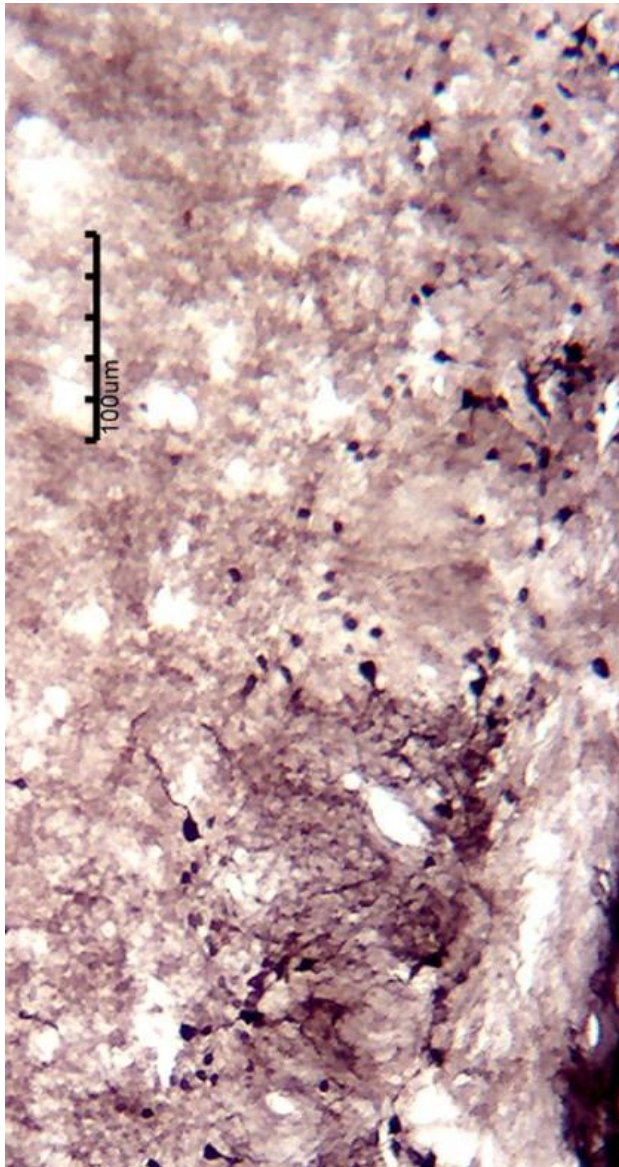

**Sham vehicle control**

Glomerular layer

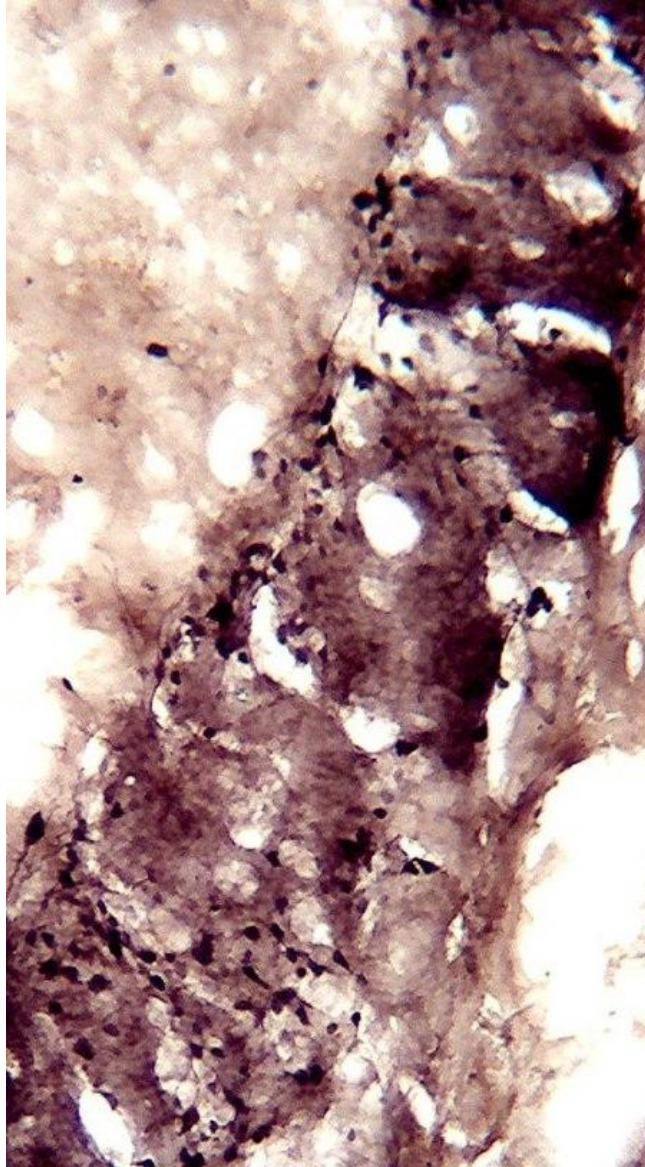

**Rotenone vehicle control**

Supplement: Supplementary file 1 [file Presentation_1.PDF]
