# Supplementary material for: Olfactory impairment in the rotenone model of Parkinson’s disease is associated with bulbar dopaminergic D2 activity after REM sleep deprivation
Source: Front Cell Neurosci. 2014 Dec 1;8:383. doi: 10.3389/fncel.2014.00383 (PMC4249459; doi:10.3389/fncel.2014.00383)
Supplement: Supplementary file 2 [file Image_1.PDF]

Supplementary material

Figure 1. ***Concentration/effect curve:***

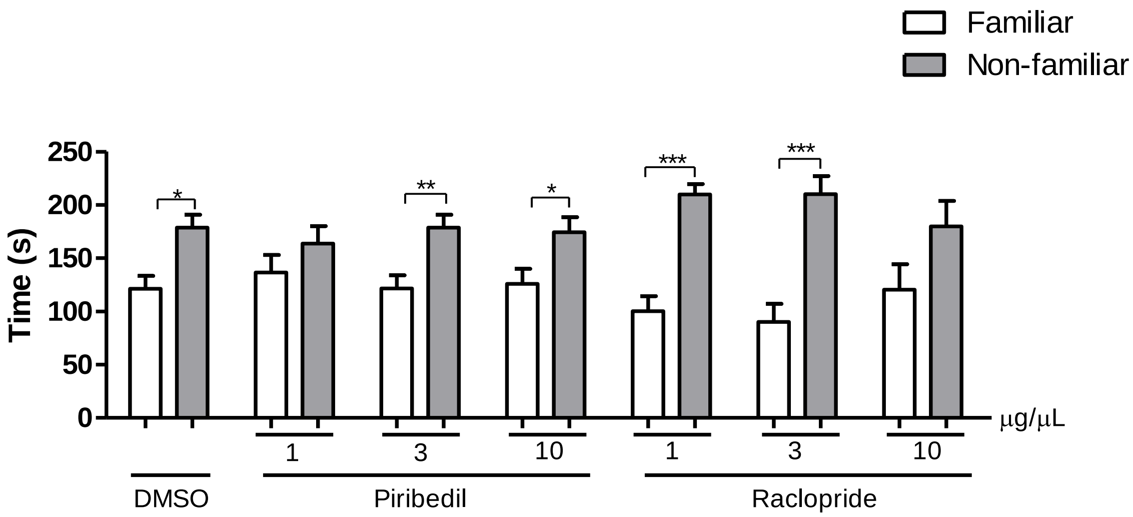

Fig. 1. Olfactory discrimination task. Time (s) spent in familiar and non-familiar compartments in the olfactory discrimination task 7 days after surgery, 120 minutes after drugs (piribedil or raclopride) or vehicle (DMSO) micro infusion, concentration 1, 3 or 10 µg/µL. The bars represent the mean ± standard error of the mean. Groups: DMSO (n=10), piribedil 1 µg/µL (n=10), piribedil 3 µg/µL (n=10), piribedil 10 µg/µL (n=10), raclopride 1 µg/µL (n=10), raclopride 3 µg/µL (n=10), raclopride 10 µg/µL (n=10), *P≤0.05, **P≤0.01, ***P≤0.001 comparing the mean time spent in the familiar and non-familiar compartments. Two-way ANOVA followed by the Bonferroni test.
