# Supplementary material for: Olfactory impairment in the rotenone model of Parkinson’s disease is associated with bulbar dopaminergic D2 activity after REM sleep deprivation
Source: Front Cell Neurosci. 2014 Dec 1;8:383. doi: 10.3389/fncel.2014.00383 (PMC4249459; doi:10.3389/fncel.2014.00383)
Supplement: Supplementary file 3 [file Image_2.PDF]

Figure 2. ***Open field test:***

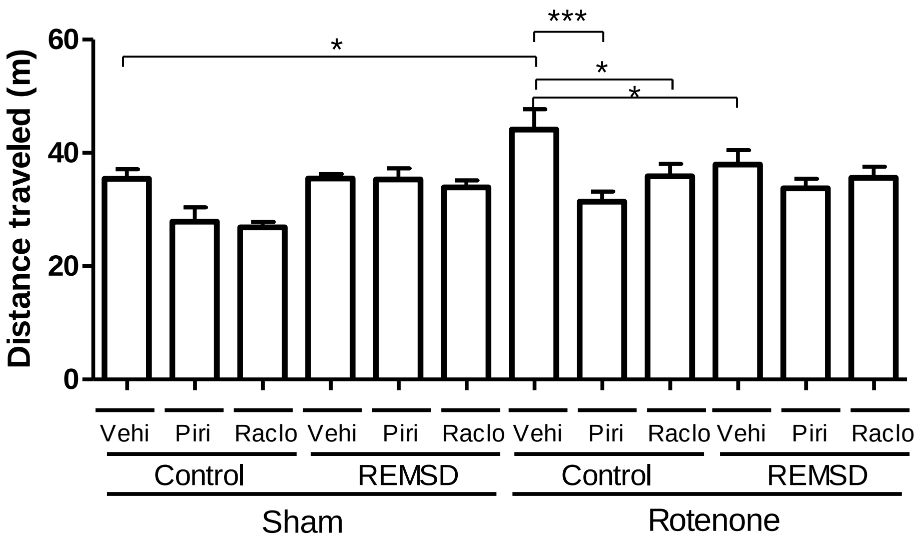

Fig. 2. Distance traveled (m) of animals in the open field test 7 days after surgery. The bars represent the mean ± standard error of the mean. Groups: DMSO (n=15/group), piribedil 3 µg/µL (n=15/group) and raclopride 10 µg/µL (n=15/group). *P≤0.05, ***P≤0.001. One-way ANOVA followed by the Newman-Keuls test.
